# Minocycline Combined with Vancomycin for the Treatment of Methicillin-Resistant Coagulase-Negative Staphylococcal Prosthetic Joint Infection Managed with Exchange Arthroplasty

**DOI:** 10.7150/jbji.43254

**Published:** 2020-04-12

**Authors:** Géraldine Bart, Valérie Zeller, Younes Kerroumi, Beate Heym, Vanina Meyssonnier, Nicole Desplaces, Marie Dominique Kitzis, Jean Marc Ziza, Simon Marmor

**Affiliations:** 1 Service de Médecine Interne et Rhumatologie, Groupe Hospitalier Diaconesses-Croix Saint-Simon, Paris, France; 2 Centre de Référence des Infections Ostéo-Articulaires Complexes, Groupe Hospitalier Diaconesses-Croix Saint-Simon, Paris, France; 3 Laboratoire des Centres de Santé et Hôpitaux d'Ile de France, Groupe Hospitalier Diaconesses-Croix Saint-Simon, Paris, France; 4 Service de Chirurgie Osseuse et Traumatologique; Groupe Hospitalier Diaconesses-Croix Saint-Simon, Paris, France; 5 Laboratoire de Microbiologie, Groupe Hospitalier Paris Saint-Joseph, Paris, France.

**Keywords:** prosthetic joint infection, methicillin-resistant *Staphylococcus*, minocycline, rifampicin

## Abstract

**Introduction:** Treatment of methicillin-resistant (MR) staphylococcal prosthetic joint infections (PJIs) remains a matter of discussion, with vancomycin-rifampin combination therapy being the preferred treatment for DAIR and one-stage exchange arthroplasty strategies. This study analyzes the outcomes of patients with chronic methicillin-resistant coagulase-negative staphylococcal PJIs treated with vancomycin-minocycline combination therapy.

**Methods:** This prospective, single center cohort study included all chronic MR coagulase-negative staphylococcal PJIs (01/2004-12/2014) treated with exchange arthroplasty and at least 4 weeks of minocycline-vancomycin. The following endpoints were considered: reinfection including relapse (same microorganism) and a new infection (different microorganism) and PJI-related deaths. Their outcomes were compared with PJIs treated with rifampin-vancomycin during the same period.

**Results:** Thirty-four patients (median age, 69 years) with 22 hip and 12 knee arthroplasty infections were included. Sixteen (47%) had previously been managed in another center. Median vancomycin MIC of strains was 3 mg/L. Nineteen underwent one-stage, 15 two-stage exchange arthroplasty. After a median [IQR] follow-up of 43 [26-68] months, 2 patients relapsed and 6 developed a new PJI. Compared to 36 rifampin-vancomycin treated PJIs, relapse- or reinfection-free survival rates didn't differ, but more new infections developed in the minocycline group (6 vs 3; *P* 0.3).

**Conclusions:** Minocycline-vancomycin combination therapy for chronic MR coagulase-negative staphylococcal PJIs seems to be an interesting therapeutic alternative.

## Background

Medical and surgical treatments of chronic prosthetic joint infection (PJI) are still a matter of debate. They combine complete removal of the infected device and effective antibiotic therapy. Staphylococci are the most frequent microorganisms isolated, with 15-50% of the isolates being methicillin-resistant 1-3. Data from randomized trials on treatment of methicillin-resistant staphylococci are lacking, and the choice of antibiotic(s) and recommendations vary according to authors 4, 5. International guidelines recommend vancomycin as the preferred treatment 4, combined with rifampin for debridement and irrigation with prosthesis retention (DAIR) and one-stage exchange arthroplasty strategies*.* Vancomycin monotherapy failed to treat experimental staphylococcal bone infection 6. Combination therapy with rifampin was the main factor associated with treatment success in humans 7, 8 and in animal models 9-11. But rifampin cannot be prescribed for all patients: indeed, resistance, allergy, drug intolerance and/or drug interactions are not uncommon.

Minocycline, a tetracycline antibiotic, is naturally effective against methicillin-resistant staphylococci, including multiresistant isolates. *In vitro*, minocycline remains effective against tetracycline-resistant isolates 12. Its bone diffusion is high and good bioavailability allows oral intake. We have used this antibiotic for many years in combination with vancomycin to treat multidrug-resistant staphylococci.

The aim of this study was to analyze the outcomes of patients treated with combination antibiotic regimen including minocycline for chronic methicillin-resistant staphylococcal PJI.

## Materials and Methods

### Study Design

This prospective cohort study was conducted in a French National Referral Center for Bone-and-Joint Infections from January 2004 to December 2014. All patients with PJIs admitted to the Center are registered in our prospective PJI cohort (NCT 01963520, NCT 02801253). Data were extracted from that database. Written informed consent was obtained from all patients and the cohort was approved by the Île-de-France Ethics Committee.

### Definition of cases and microbiology

All patients treated for chronic, i.e., lasting >1 month, methicillin-resistant staphylococcal PJIs who underwent 1- or 2-stage exchange arthroplasty were eligible. To be included in the minocycline group, patients had to be ≥18 years, and receive at least 4 weeks of first-line antibiotics combining oral minocycline and continuous intravenous (IV) vancomycin. PJI was defined 13 as isolation of the same microorganism from ≥2 cultures of preoperative joint-fluid and/or intraoperative tissue specimens plus ≥1 of the following criteria: a sinus tract communicating with the prosthesis, local inflammatory signs (swelling, warmth, erythema), C-reactive protein (CRP) >5 mg/L and/or radiological parameters (i.e., periosteal bone formation, subchondral osteolysis) of infection.

Patients with polymicrobial PJIs, including microorganism(s) other than staphylococci, were not included.

Finally, patients with methicillin-resistant *S. aureus* were non-included, because they were very few (n=3).

Preoperative joint aspirates and intraoperative samples were handled as previously described 3*.* Drug-resistance patterns were determined with the disk-diffusion method for all specimens and for each colony with a different morphology. Vancomycin MICs were determined with E-test (BioMérieux, Marcy l'Étoile, France). When a given patient had several different isolates identified, we retained the most resistant staphylococcal strain for treatment and description.

### Treatment modalities

All minocycline group patients received initial combination antibiotic therapy with high-dose continuous IV vancomycin and oral minocycline for 4-6 weeks, followed by 6-8 weeks of an oral regimen. Vancomycin treatment modalities and drug monitoring have previously been described 14. A continuous serum vancomycin concentration of 30-35 mg/L was targeted. The patients didn't receive initial empirical large spectrum antibiotic therapy, as preoperative joint aspiration confirmed PJI with methicillin-resistant *Staphylococcus*.

Reasons using minocycline instead of rifampin-combination therapy were rifampin resistance, drug interaction, previous intolerance, reported allergy to rifampin, or previous treatment failure with rifampin.

Minocycline was taken orally: 100 mg thrice daily for patients weighing <90 kg and 200 mg twice a day for those >90 kg. To avoid its malabsorption, all concomitant medications associated with decreased drug absorption (iron, antacids containing aluminium or magnesium salts.) were stopped. Treatment compliance and gastrointestinal tolerance were monitored throughout the entire treatment duration.

Adverse drug-reaction severity was assessed according to the Common Terminology Criteria for Adverse Events (CTCAE) 15. Antibiotic withdrawal was decided by the treating physician when severe side effects occurred (≥ CTCAE grade 2).

One-stage exchange arthroplasty was performed, provided that no bone reconstruction was necessary and that the microorganism had been isolated from pre-operative joint aspirates 16. The remaining patients underwent 2-stage exchange arthroplasty. No antibiotic-loaded bone cement was used, neither in the spacer, nor to fix the prosthesis.

### Follow-up and outcomes

Patients were discharged at the end of IV antibiotic therapy and then seen as outpatients, at 3, 6, 12 and 24 months, and then every 2 years. For patients not seen for >2 years, they or their primary physicians were contacted by telephone. Outcome analysis required a minimum of 2 years post-surgery to the last follow-up visit. The following endpoints were considered: reinfection including relapse with the same microorganism as the initial PJI or a new infection with a microorganism different from the initial PJI, and PJI-related death (infection- or treatment-related).

### Statistical analyses

The primary outcome of interest was the cumulative probability of reinfection, i.e., relapse or new infection.

Qualitative variables are expressed as number (%) and compared using χ^2^ tests. Quantitative variables are expressed as median [interquartile range; IQR], first assessed for normality and then compared with either Student or Mann-Whitney *U*-test. The relapse- and reinfection-free survival rate was analyzed using Kaplan-Meier method and expressed as the percentage ± standard deviation. The Mantel-Cox log-rank test was used to test the between-group survival-distribution difference. A *P* value < 0.05 was considered significant. All statistical tests were performed with SPSS.20 software.

### Comparative study

To assess minocycline-combination-therapy efficacy and tolerance, we compared outcomes of these patients to those receiving initially 4-6 weeks of continuous IV vancomycin and rifampin-combination therapy, followed by 6-8 weeks of an oral regimen and managed during the same period. These patients were defined as the rifampin group. We chose rifampin-combination therapy as comparator according to IDSA guidelines, as it is the preferred therapy for methicillin-resistant staphylococcal PJIs treated with DAIR and one-stage implant exchange 4.

## Results

### Population

**Figure 1:**
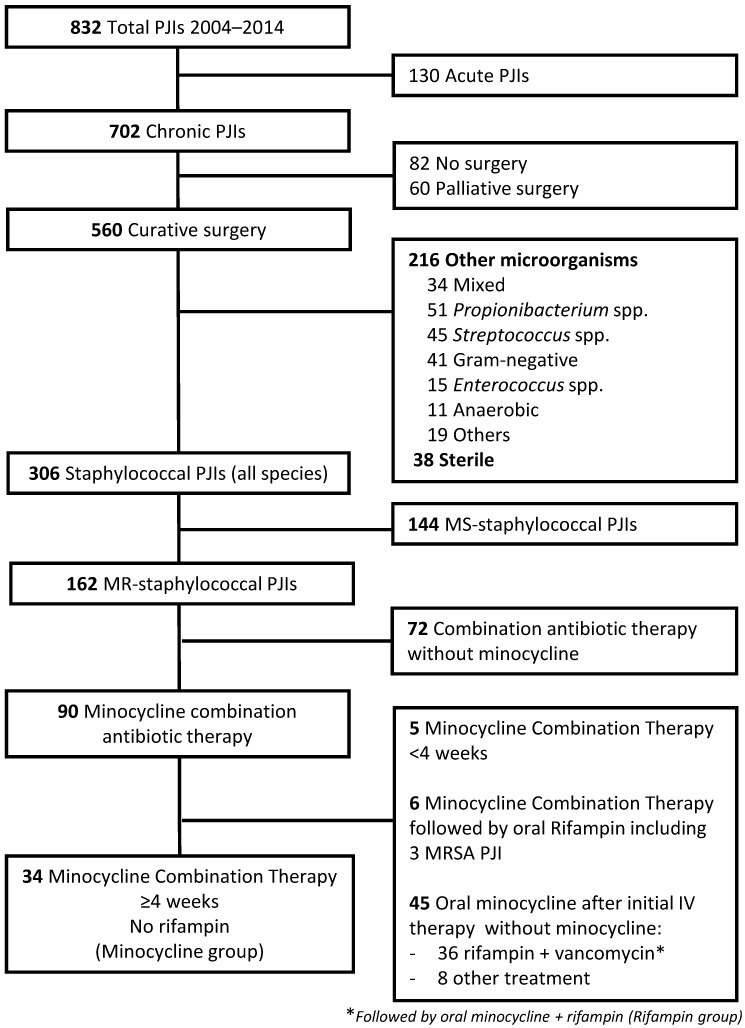
Flow-chart of the study population

During the study period, among 832 PJIs managed in our Center, 90 (10.8%) were chronic methicillin-resistant staphylococcal PJIs, who underwent one or two-stage exchange arthroplasty and received minocycline-combination therapy (Figure 1).

We excluded 5 patients who received <4 weeks of initial minocycline combination therapy, and 45 others who took only oral minocycline, after initial IV combination therapy with vancomycin-rifampin (n=36, all these patients were included in the rifampin comparator group), vancomycin-fusidic acid (n=2), vancomycin-fosfomycine (n=2), vancomycin-gentamicin (n=2), or vancomycin (n=1) or linezolid monotherapy (n=1).

To avoid confusion biases, 6 minocycline patients who had received rifampin during the oral therapy, were excluded from the analysis. Three methicillin-resistant *S. aureus* PJI were included in this group.

Finally, 34 (5%) patients were included in the minocycline group. Baseline characteristics of the patients are reported in Table 1.

Reasons not to use initial rifampin combination therapy were rifampin resistance in 26, potential drug interaction in 1, previous treatment failure with rifampin in 2, drug intolerance in 2, allergy in 1 and not determined in 2 patients.

### Microbiology

The staphylococcal species isolated in the minocycline group are shown in Table 1. The most frequently isolated microorganism was methicillin- resistant *S. epidermidis* (91%). Vancomycin MIC was >2 mg/L for more than two-thirds (n=24) of the isolates. Resistance patterns to other antibiotics are reported in Table 2. Six patients harbored strains resistant to all the other usual antistaphylococcal agents. Eight patients' isolates were tetracycline-resistant but remained minocycline-susceptible.

### Antibiotics and Surgery

Durations of IV and total antibiotics, and surgical interventions in the minocycline group are detailed in Table 3.

The initial IV antibiotic therapy was followed by 6-8 weeks of an oral regimen with minocycline monotherapy, except 7 who received linezolid (n=2) or combination therapy with minocycline and clindamycin (n=5).

All the patients received minocycline 100 mg, thrice daily, except an 88-year old woman given 100 mg twice daily and 5 others taking 200 mg twice daily.

### Outcomes

**Figure 2:**
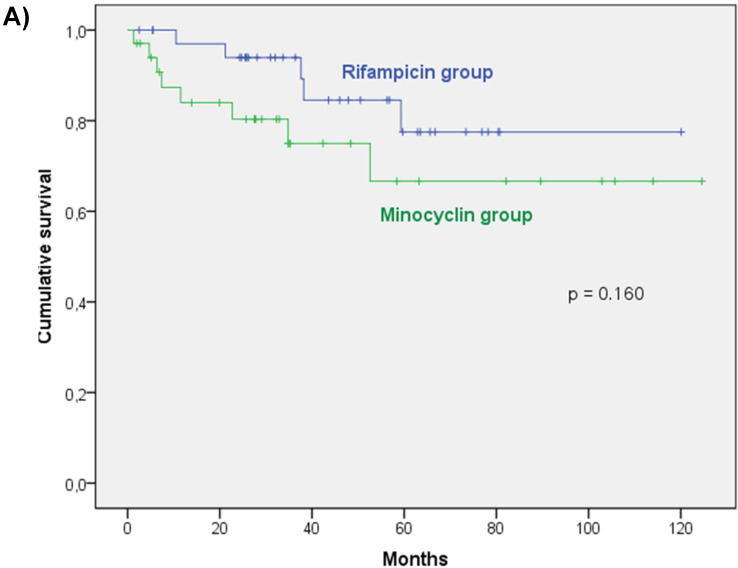

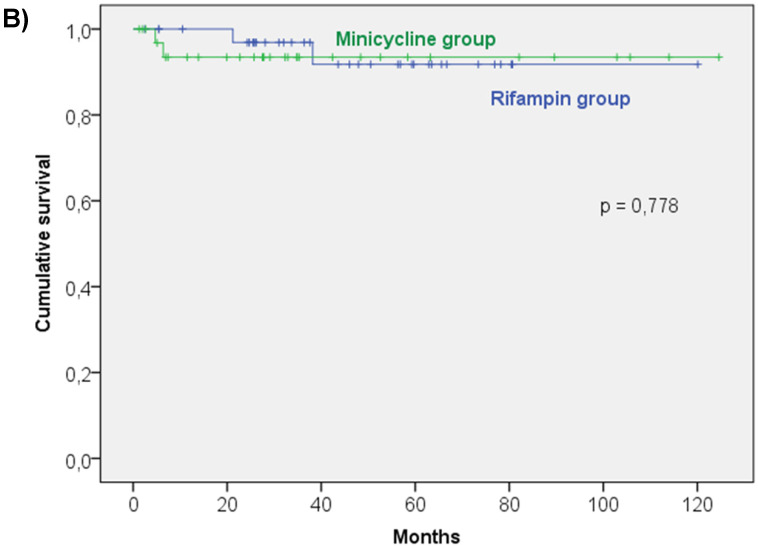
Kaplan Meier survival curve free of reinfection (Fig. 2A) and relapse (Fig. 2B) in the minocycline and rifampin combination therapy groups

Median [IQR] follow-up was 43 26-28 months. One patient was lost to follow-up before 2 years. No PJI-related deaths occurred in our cohort. Reinfection and relapse-free survival rates are shown in Figure 2A and 2B.

Only 2 patients with methicillin-resistant *Staphylococcus epidermidis* knee-arthroplasty infections relapsed. Both had undergone 2 or 3 prior surgeries for their PJIs. Their staphylococci were only susceptible to cyclines, linezolid and glycopeptides (vancomycin MIC 2 mg/L). One underwent one-stage, the other two-stage exchange arthroplasty. Both received vancomycin-minocycline for 6 weeks, followed by minocycline for further 6 weeks and relapsed 1 month after stopping antibiotics. No minocycline resistance was observed. One patient's vancomycin MIC increased from 2 to 4 mg/L.

Six patients with prosthetic hip infections developed new infections: 4 were late chronic infections that developed 2-4 years after the first PJI and were caused by *Pseudomonas aeruginosa*, *Enterobacter cloacae*, *Cutibacterium acnes* or polymicrobial flora, respectively. Acute hematogenous PJIs occurred in 2 other patients one year after their first PJIs and were due to susceptible* S. aureus* and *Proteus mirabilis*.

Three patients experienced minocycline adverse events, necessitating drug withdrawal for one with thrombopenia and another with cholestatic hepatitis. Three patients developed grade 2 renal insufficiency attributed to vancomycin.

Five patients initially treated with vancomycin-minocycline were not included in the study because they took minocycline less than 4 weeks (see Figure 1). Reasons for minocycline discontinuation after 3-21 days were an adverse event for 3 patients (rash, persistent vomiting, renal toxicity for which vancomycin was also stopped). If we take these 3 additional patients into account, adverse events occurred in 9 (21%), requiring drug withdrawal for 4 (9%) of the 43 patients.

### Comparative Analysis

Thirty-six patients received initial vancomycin-rifampin combination therapy. Their characteristics, shown in Tables 1 and 3, differed significantly from those of the minocycline-treated patients, with the latter having more prosthetic knee infections (12 vs 5; *P* = 0.052), but fewer hip infections (22 vs 31; *P* = 0.052), their isolated strains had higher vancomycin MICs (3 vs 2 mg/L; *P* = 0.001), and with a trend towards more patients having undergone 3 or more surgeries for their PJIs (6 vs 1; *P* = 0.052). Two-stage exchange arthroplasty was done more frequently in the minocycline group, without reaching significance (15 vs 8; *P* = 0.075). Although more new infections occurred in the minocycline group (6 vs 3; *P* 0.3), neither relapse- nor reinfection-free survival rates differed significantly between the 2 groups (Figure 2, Table 3). Adverse events were more frequently gastrointestinal symptoms in the rifampin group, without reaching significance (Table 3).

## Discussion

Herein, we reported our results with minocycline-combination therapy to treat chronic methicillin-resistant coagulase-negative staphylococcal PJIs over 10 years. We identified 34 patients whose initial regimen combined minocycline with continuous high-dose IV vancomycin. These patients mostly had complex “difficult-to-treat” PJIs, and we can conclude that this regimen was effective. Indeed, only 2 patients (6%) relapsed and 80% of the patients were infection-free at 2 years of follow-up, even though 47% of them had already undergone at least 1 surgery for their PJIs and multiresistant staphylococci prevented the use of "gold standard" antibiotics, e.g. fluoroquinolones or rifampin 4, 5.

To enhance the message of these outcomes, we compared the minocycline treated group to patients treated during the same time period with vancomycin-rifampin, the preferred treatment for methicillin-resistant staphylococcal PJI 4. Minocycline-combination-treated patients appeared to have more complex infections. Indeed, they had more frequently had previous surgery for their PJIs, their staphylococcal strains had higher vancomycin MICs and more of them had knee infections. Two-stage replacement was also more often performed. Although relapse rates did not differ between the 2 groups, more new infections occurred in the minocycline group, but the difference was not significant. That higher new infection rate is probably due to their more frequent knee infections and two-stage exchange arthroplasty, known to have higher new infection rates in our experience, and their more complex PJIs.

Minocycline has been used for many years to treat multiresistant staphylococcal infections 17, 18, especially methicillin-resistant *S. aureus* (MRSA) skin-and-soft-tissue infections 19, 20. Raad et al. 21 showed that minocycline has very good *in vitro* activity against MRSA embedded in biofilm. Our Referral Center's long experience using minocycline started in 1984, following Biddle's presentation at the 24^th^ ICAAC, to treat methicillin-resistant staphylococcal bone-and-joint infections, although further and larger data in humans are lacking. Pertinently, minocycline was always used in combination with vancomycin during the first weeks of treatment.

Among the tetracycline-family members, minocycline has higher *in vitro* susceptibility rates than tetracycline and doxycycline 22, 23. Cunha reported several cases of MRSA skin-and-soft-tissue infections unresponsive to doxycycline that responded rapidly to minocycline 20. Yuk et al. 23 described 21 minocycline-treated patients with various staphylococcal infections, 15 being tetracycline-resistant. Eight of our minocycline-treated patients harbored a tetracycline-resistant *Staphylococcus* strain susceptible to minocycline; none of them relapsed. That finding highlights minocycline efficacy even against tetracycline- or doxycycline-resistant strains 22. Resistance to tetracyclines is mostly due to protein-efflux pumps, a molecule-specific mechanism 24. Gram-positive microorganisms contain primarily the *tet*(K) and *tet*(L) genes coding for drug-efflux pumps that confer resistance to tetracycline and doxycycline, but not minocycline or tigecycline 25.

Other important advantages of minocycline are its low cost, its oral administration and good bone diffusion. To treat these complex bone and joint infections we used higher minocycline dosage than recommended in order to reach effective minocycline bone concentrations. Among our patients, we observed high minocycline bone penetration in 6 (unpublished data), with bone minocycline concentrations well above those of serum (median bone concentration, 7.85 [2.2-46.3] µg/g; median trough serum concentration, 1.5 [0.3-3.6] µg/mL). No study on minocycline bone diffusion has been published yet and data on diffusion of other tetracyclines are scarce 26, 27.

All our patients received vancomycin-combination therapy, even those treated with two-stage exchange. IDSA guidelines recommend combination therapy with rifampin only in case of implant retention (DAIR) or one-stage exchange arthroplasty. But experiences in animals showed that vancomycin monotherapy failed to sterilize staphylococcal bone infections 6. Data in humans on this question are scarce 28. These results lead us choosing combination therapy in all patients with methicillin-resistant bone and joint infections. Minocycline-combination therapy with rifampin has been described 17, 29 to treat various severe methicillin-resistant *S. aureus* infections. Data on other combination therapies are lacking.

Tigecycline, a glycylcycline antibiotic derived from minocycline, is effective against a broad-spectrum of bacteria, including methicillin-resistant staphylococci. Success rates of 76-85% after tigecycline treatment of bone-and-joint infections in 2 case series have been reported 30, 31. Most of those patients had infections with multiresistant Enterobacteriaceae and 20-30% had staphylococcal infections. Despite those findings, we chose to continue to use minocycline because of its good efficacy, lower cost and oral administration.

Drug tolerance in our study was acceptable, despite the use of higher dosages. Adverse event and withdrawal rates in the minocycline treated group are lower than those reported for other anti-staphylococcal antibiotics 32-34.

Our single center, observational, comparative study, has several limitations. First, all the patients received combination therapy with vancomycin, limiting the evaluation of minocycline efficacy alone. However, previous studies, especially animal models, showed that vancomycin monotherapy cannot cure chronic staphylococcal osteomyelitis 6. We wouldn't recommend minocycline monotherapy to treat these complex multiresistant infections, although it is not clear if combination therapy is required for the treatment of PJI managed by implant removal. It has been shown that minocycline resistance can develop rapidly 24. Second, the number of patients included is limited because we focused specifically on complex PJIs treated with exchange arthroplasty. One can wonder whether the absence of outcome differences between the vancomycin-minocycline- and vancomycin-rifampin-treated groups might be due to a lack of power. We don't have enough hard clinical evidence to show that minocycline-vancomycin is as effective as rifampin-vancomycin therapy. Our results have to be confirmed by larger studies. Third, we didn't include neither *S. aureus* infections, because they were only very few in the cohort and *S. aureus* is a notably different organism to treat, nor polymicrobial infections with non-staphylococcal species to avoid confusing results. Fourth, the study was not randomized and the risk of bias exists. Indeed, some patients' characteristics differ between the groups, as already discussed. Finally, to analyze minocycline efficacy, we excluded patients who had received it for less than 4 weeks, including those who stopped minocycline because of adverse events, and those who received rifampin during oral treatment. But to examine tolerance more thoroughly, we separately analyzed the patients with less than 4 weeks of minocycline and addressed those observations in the discussion of tolerance.

In conclusion, minocycline-vancomycin combination therapy to treat complex chronic methicillin-resistant coagulase-negative staphylococcal PJIs managed with exchange arthroplasty achieved 80% favorable outcomes at 2 years. Minocycline appears to be an effective therapeutic alternative for these difficult-to-treat infections, especially when rifampin cannot be used. More data from large prospective randomized trials are necessary to confirm our preliminary observations.

## Figures and Tables

**Table 1:** Clinical and microbiological characteristics at baseline of patients treated for PJI with minocycline and rifampin combination therapy

	Minocycline group (n=34)	Rifampin group (n=36)	p value
Age, years, Mean [IQR*]	69 [62-73]	66 [61-76]	0,972
Male, n (%)	24 (71)	22 (61)	0,457
Body Mass Index, kg/M2, median [IQR]	28 [24-31]	29 [24-31]	0,707
ASA score > 2 n, (%)	11 (32)	10 (28)	0.6
**Comorbidities**			
Diabetes mellitus, n (%)	5 (15)	4 (11)	0,731
Neoplasia, n (%)	4 (12)	5 (14)	1.000
Immunocompromized (any cause), n (%)	6 (18)	9 (25)	0,564
Renal insufficiency (CK <60mL/min), n (%)	7 (21)	8 (22)	1.000
Active smoker, n (%)	5 (15)	7 (19)	0,749
Active alcohol abuse, n (%)	2 (6)	4 (11)	0,671
**Infection characteristics**			
Prosthetic hip infection, n (%)	22 (65)	31 (86)	0,052
Prosthetic knee infection, n (%)	12 (35)	5 (14)	0,052
Duration of symptoms before surgery in our center, months, median [range]	15 [10-42]	11 [8-22]	0.390
Duration between last "clean" surgery and surgery in our center, months, median [IQR]	28 [16-55]	17 [9-47]	0,173
Previous management of PJI before admission in our center, n (%)	16 (47)	14 (39)	0.630
One previous surgery for PJI before admission in our center, n (%)	6 (18)	9 (25)	0,564
Two previous surgery for PJI before admission in our center, n (%)	4 (12)	4 (11)	1.000
≥ 3 previous surgery for PJI before admission in our center, n (%)	6 (18)	1 (3)	0,052
**Microorganism**			
Staphylococcus			
*S. epidermidis* n (%)	31 (91)	29 (81)	0,308
Other coagulase negative *Staphylococcus*, n	1	0	
Mixed *Staphylococcus* infection, n (%)	2 (6)	7 (19)	0,152
Vancomycin MIC**, median [IQR]	3 [2-4]	2 [2-3]	0,001
Vancomycin MIC >2, n (%)	24 (71)	12 (33)	0,007

*IQR = interquartile range. **MIC = Minimal Inhibitor Concentration.

**Table 2:** Antibiotic resistance of the strains of 34 patients with MR staphylococcal PJI treated with minocycline combination therapy Minocycline group)

	n	%
**minocycline**	0	0
**tetracycline**	8	24
**Other antibiotics**		
gentamicin	27	79
erythromycin	24	71
clindamycin	14	41
rifampin	26	76
fusidic acid	27	79
quinolones	30	88
cotrimoxazole	26	76
**Resistance to all these molecules except minocyclin**	6	18

**Table 3:** Duration of antibiotic therapy, surgical treatment and outcome of PJI treated with minocycline and rifampicin combination therapy

		Minocycline group n=34	Rifampin group n=36	p value
**Antibiotic therapy**				
Duration of IV therapy, days, median [IQR*]		42 [40-44]	42 [40-43]	0,474
Duration of total antibiotic therapy, days, median [IQR]		85 [84-90]	84 [84-85]	0,085
**Surgical treatment**				
One-stage replacement		19 (56)	28 (78)	0,075
Two-stage replacement		15 (44)	8 (22)	0,075
**Outcome**				
Median duration of follow-up, months, median [IQR]		43 [26-68]	49 [27-72]	0,632
Re-infection, n (%)		8 (24)	5 (14)	0,365
	Relapse, n (%)	2 (6)	2 (5)	1.000
	New infection, n (%)	6 (18)	3 (8)	0.300
**Adverse events due to minocyclin**		3** (10)	9** (25)	0,112
	Skin signs, n (%)	0	1	1.000
	Gastrointestinal symptoms, n (%)	1	4	0,358
	Cytopenia, n (%)	1	1	1.000
	Hepatitis, n (%)	1	3	0.615
Drug withdrawal after adverse event		2	4	0,674

*IQR = interquartile range. **: one patient had concomittantly hepatitis and skin signs, the other hepatitis and gastrointestinal symtptoms.
